# DOES THE PATIENT HEALTH QUESTIONNAIRE MEASURE MOOD-RELATED QUALITY OF LIFE IN NURSING FACILITY RESIDENTS?

**DOI:** 10.1093/geroni/igz038.574

**Published:** 2019-11-08

**Authors:** Weiwen Ng, Yinfei Duan, Tetyana P Shippee

**Affiliations:** 1 University of Minnesota, Minneapolis, Minnesota, United States; 2 School of Nursing, University of Minnesota, Minneapolis, Minnesota, United States

## Abstract

The Patient Health Questionnaire-9 (PHQ-9) is a depressive symptom questionnaire administered to nursing facility (NF) residents in the Minimum Data Set (MDS). Does the PHQ-9 measure mood-related aspects of quality of life (QoL)? We assessed the PHQ-9’s convergent validity with negative and positive mood items from Minnesota’s QoL survey, which is administered annually to a random sample of residents. We also examined if scores on both instruments were associated with various psychiatric diagnoses on the MDS. Using item response theory (IRT) models, we estimated that depressive symptoms (PHQ-9) had a correlation of 0.546 with negative mood and -0.425 with positive mood. With explanatory IRT modeling, we estimated that diagnoses of anxiety, depression, and bipolar disorder were respectively associated with 0.261, 0.339, and 0.301 (all p < 0.001) standard deviation increases in (SD) depressive symptoms, and with 0.235, 0.261, and 0.306 SD increases in negative mood (all p < 0.001), thus indicating convergent validity. For positive mood, depression and bipolar disorder had associations of similar magnitude as the other two constructs. However, anxiety disorders were not associated with lower positive mood (-0.014 SD, p = 0.636). Thus, the PHQ-9 can measure mood-related aspects of QoL. However, the PHQ-9 appears to be sensitive to relatively serious depression, whereas the Minnesota items are more sensitive to lower levels of negative mood. Also, the PHQ-9 does not measure positive mood directly. Thus, the PHQ-9 is a more limited measure of mood-related QoL than the Minnesota items.

